# Synthetic MRI demonstrates prolonged regional relaxation times in the brain of preterm born neonates with severe postnatal morbidity

**DOI:** 10.1016/j.nicl.2020.102544

**Published:** 2020-12-24

**Authors:** Tim Vanderhasselt, Roya Zolfaghari, Maarten Naeyaert, Jeroen Dudink, Nico Buls, Gert-Jan Allemeersch, Hubert Raeymakers, Filip Cools, Johan de Mey

**Affiliations:** a Department of Radiology, Vrije Universiteit Brussel, Universitair Ziekenhuis Brussel, Brussels, Belgium; b Department of Neonatology, Vrije Universiteit Brussel, Universitair Ziekenhuis Brussel, Brussels, Belgium; c Department of Neonatology, Wilhelmina Children's Hospital, University Medical Center Utrecht, Utrecht, The Netherlands; d Brain Center University Medical Center Utrecht, Utrecht, The Netherlands

**Keywords:** PLIC, posterior limb of the internal capsule, PMA, postmenstrual age, TEA, term equivalent age, ANCOVA, analysis of covariance, MANTiS, Morphologically Adaptive Neonatal Tissue Segmentation, Quantitative MRI, Brain, Infant, Newborn, Premature, Biomarkers

## Abstract

**Background:**

To identify preterm infants at risk for neurodevelopment impairment that might benefit from early neurorehabilitation, early prognostic biomarkers of future outcomes are needed.

**Objective:**

To determine whether synthetic MRI is sensitive to age-related changes in regional tissue relaxation times in the brain of preterm born neonates when scanned at term equivalent age (TEA, 37–42 weeks), and to investigate whether severe postnatal morbidity results in prolonged regional tissue relaxation times.

**Materials and methods:**

This retrospective study included 70 very preterm born infants scanned with conventional and synthetic MRI between January 2017 and June 2019 at TEA. Infants with severe postnatal morbidity were allocated to a high-risk group (n = 22). All other neonates were allocated to a low-risk group (n = 48). Linear regression analysis was performed to determine the relationship between relaxation times and postmenstrual age (PMA) at scan. Analysis of covariance was used to evaluate the impact of severe postnatal morbidity in the high-risk group on T1 and T2 relaxation times. Receiver operating characteristic (ROC) curves were plotted and analysed with area under the ROC curve (AUC) to evaluate the accuracy of classifying high-risk patients based on regional relaxation times.

**Results:**

A linear age-related decrease of T1 and T2 relaxation times correlating with PMA at scan (between 37 and 42 weeks) was found in the deep gray matter, the cerebellum, the cortex, and the posterior limb of the internal capsule (PLIC) (p < .005 each), but not in the global, frontal, parietal, or central white matter. Analysis of covariance for both risk groups, adjusted for PMA, revealed significantly prolonged regional tissue relaxation times in neonates with severe postnatal morbidity, which was best illustrated in the central white matter of the centrum semiovale (T1 Δ = 11.5%, T2 Δ = 13.4%, p < .001) and in the PLIC (T1 Δ = 9.2%, T2 Δ = 6.9%, p < .001). The relaxation times in the PLIC and the central white matter predicted high-risk status with excellent accuracy (AUC range 0.82–0.86).

**Conclusion:**

Synthetic MRI-based relaxometry in the brain of preterm born neonates is sensitive to age-related maturational changes close to TEA. Severe postnatal morbidity correlated with a significant delay in tissue relaxation. Synthetic MRI may provide early prognostic biomarkers for neurodevelopment impairment.

## Highlights

• Early biomarkers are needed to identify preterm infants at risk for neurodevelopment impairment.
• Synthetic MRI-based relaxometry is sensitive to age-related maturational changes.
• Severe postnatal morbidity results in significantly prolonged regional tissue relaxation times.
• Early myelinating structures appear to be more susceptible to brain injury.
• Synthetic MRI may provide early prognostic biomarkers for neurodevelopment impairment.

## 1 Introduction

Severe white matter injuries in very preterm-born neonates have declined substantially in the last decades owing to improved neonatal care (Larroque et al., 2008). Nevertheless, the immature preterm brain remains particularly vulnerable to subtle forms of injury and disrupted brain maturation that are associated with cognitive, sensorimotor, and behavioral disabilities later in life (Larroque et al., 2008). Unfortunately, those disabilities can often not be reliably diagnosed before the age of 3–5 years while neuroplasticity, and consequently neurorehabilitation potential, is at its highest in the first few years of life (Johnston, 2009). Therefore, it is essential to identify children at risk of disability at a very early stage in order for them to benefit from early neurorehabilitation (Parikh, 2016, Spittle et al., 2015). Most research so far studied the relationship between MRI findings at the term equivalent age (TEA) and long-term neurodevelopmental outcomes of very preterm infants. Therefore, many centers, including ours, standardized neonatal MRI around this period (Plaisier et al., 2014). However, the predictive value of conventional MRI is still limited because it is not sensitive enough to quantify microstructural brain injury (Counsell, 2003). The availability of quantitative biomarkers that are predictive of future outcomes is therefore much needed.

Measurements of longitudinal (T1) and transverse (T2) relaxation times are particularly suited for studying neonatal brain maturation because they reflect microstructural changes in bulk water content, compartmentalization, and macromolecules that are associated with early brain maturation and myelination (Deoni et al., 2012). Relaxation times decrease with age and are more sensitive to normal and abnormal brain maturation than visual assessment (Counsell et al., 2003, Ferrie et al., 1999, Hagmann et al., 2009, Knight et al., 2018, Leppert et al., 2009, Maitre et al., 2014, Nossin-Manor et al., 2013, Schneider et al., 2016, Williams et al., 2005). Studies of relaxation times at TEA in very preterm born children are however rare (Counsell et al., 2003, Knight et al., 2018, Maitre et al., 2014, Schneider et al., 2016, Williams et al., 2005), and the relationship with postnatal morbidity or outcome has to our knowledge not been investigated.

Synthetic MRI is an emerging and commercially available technique that combines conventional high spatial resolution MRI images with quantitative T1 and T2 relaxometry, and volumetry, based on a 6-minute multi-delay, multi-echo acquisition (Warntjes et al., 2008). Previous studies reported that in preterm neonates, the image quality, accuracy of lesion detection, and automated volumetric measurements of synthetic MRI were as good as with conventional MRI (Schmidbauer et al., 2020, Vanderhasselt et al., 2020). In addition, synthetic MRI proved to be more sensitive to delayed myelination compared to conventional MRI sequences (Schmidbauer et al., 2019).

The objectives of this study were to investigate in a large cohort of very preterm born children (a) whether synthetic MRI-based regional relaxation times correlate with age-related maturation when scanned close to TEA, and (b) whether preterm infants at high-risk of neurodevelopmental impairment show prolonged regional tissue relaxation times compared to low-risk infants.

## 2 Methodology

### 2.1 Patients

The current retrospective study was conducted at the Departments of Radiology and Neonatology in our institution after approval by the local Institutional Review Board (B.U.N. 143201835432). Informed consent was waived due to the retrospective nature of the study. The study included 91 very preterm (born at ≤ 32 weeks of gestation) and extremely preterm (born ≤ 28 weeks) infants (Moutquin, 2003) who were scanned between January 2017 and June 2019 and for which both conventional 3D T2WI MRI and synthetic MRI were performed as part of their routine neonatal MRI exam at TEA (range 37 to 42 weeks). In a prior study, we reported the diagnostic synthetic image quality and accuracy of automated volumetry for this cohort (Vanderhasselt et al., 2020).

Infants with severe postnatal morbidity are at a higher risk of later displaying neurodevelopmental disabilities than infants without severe postnatal morbidity (Larroque et al., 2008). The neonates were therefore allocated to a high-risk group when they presented with at least one of the following characteristics of severe postnatal morbidity: severe brain injury on MRI (cystic periventricular leukomalacia or germinal matrix hemorrhage grade 3 or 4), surgical intervention for necrotizing enterocolitis or patent ductus arteriosus, treatment with postnatal steroids, prolonged mechanical ventilation > 8 days, or bronchopulmonary dysplasia defined as oxygen need at 36 weeks PMA (Ehrenkranz et al., 2005). All other neonates without severe postnatal morbidity were allocated to the low-risk group.

### 2.2 Magnetic resonance imaging

All infants were scanned on the same 3 T MRI system (Ingenia, Philips Medical Systems, The Netherlands) in a supine position using a 16-channel head coil. They were placed in a MedVac vacuum immobilization system (CFI Medical Solutions Incorporation, Fenton, Michigan) and sedated with chloral hydrate 25–50 mg/kg. Neonatal ear covers (MiniMuffs, Natus Medical, San Carlos, California) and headphones were both used for ear protection.

**Fig. 1:**
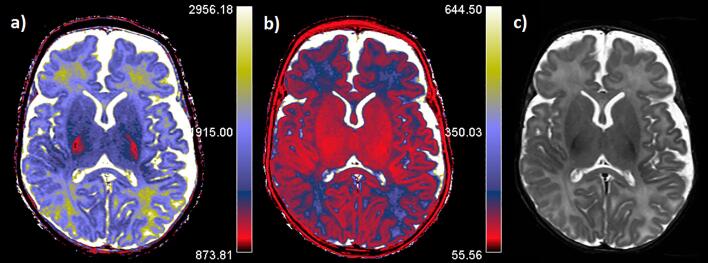
Representative sample of synthetic MRI-based T1 (a) and T2 (b) relaxation time maps. (c) Synthetic reconstructed T2 STIR image is shown at the same level.

The routine clinical scan protocol included conventional 3D T1WI fast spoiled gradient echo, 3D T2WI TSE, susceptibility-weighted imaging, DWI, Multishell DTI, and synthetic multi-delay multi-echo. The total scan time was approximately 31 min. Detailed acquisition parameters are presented in inline SupplementaryTable 1. SyMRI software version 11.1 (SyntheticMR AB, Linköping, Sweden) was used to calculate T1 and T2 relaxation maps and to reconstruct synthetic T1WI, T1 SPIR, T2WI, and T2 STIR images for diagnostic evaluation (Warntjes et al., 2008). Fig. 1 shows a representative example of T1 and T2 relaxation maps.

### 2.3 MRI data analysis

Automated tissue classification in cortical gray matter, deep gray matter, global white matter, and cerebellum was performed with MANTiS (Morphologically Adaptive Neonatal Tissue Segmentation) using the conventional 3D T2WI datasets (Beare et al., 2016). MANTiS is a state of the art open-source software developed for MRI tissue classification of the neonatal brain. It was created as a toolbox for SPM12 (2016, Wellcome Department of Imaging Neuroscience, London, UK) and is available for download at http://developmentalimagingmcri.github.io/mantis. Before tissue classification, brain extraction was achieved using the watershed-based method built-in to MANTiS.

The synthetic T2W images were automatically coregistered to the conventional 3D-T2 using SPM12 and MATLAB R2016b (The Mathworks, Natick, MA, USA), and the same transformation was applied to the T1 and T2 relaxation maps in order to automatically extract regional relaxation parameters of the tissue segmentation that were generated by MANTiS.

**Fig. 2:**
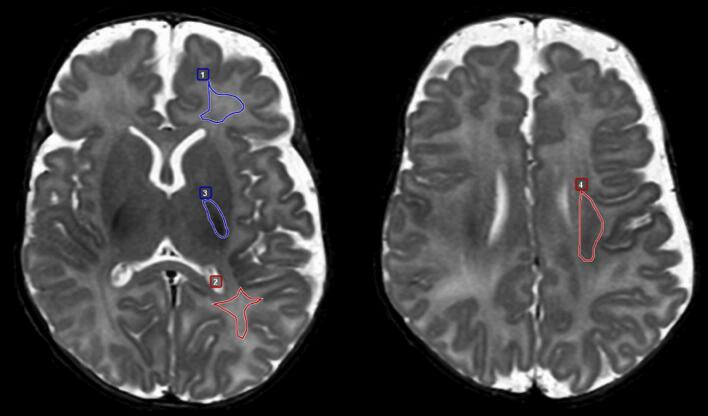
Synthetic reconstructed T2 STIR showing manual delineated regions of interest: (1) frontal white matter, (2) parietal white matter, (3) posterior limb of the internal capsule, (4) central white matter of the centrum semiovale.

MANTiS does not segment white matter into subregions. Therefore, additional manual regions of interest were delineated in SyMRI on the synthetic T2 STIR images in the frontal, parietal, and central white matter of the centrum semiovale and in the posterior limb of the internal capsule (PLIC) (Fig. 2). Focal white matter lesions and cysts were carefully excluded from the regions of interest. Mean T1 and T2 relaxation values in these regions were automatically provided by the SyMRI software. To avoid user bias, all regions of interest were delineated by the same pediatric neuroradiologist (TV), who has 13 years of experience in neonatal MRI and who was blinded to the subgroups. The measurements were performed a second time for all patients (n = 70) with an interval of four months by the same radiologist to calculate intra-rater variability. A fellow in neuroradiology (GJA) and a medical master student (RZ) both independently performed measurements once on a randomized subset of 35 patients to assess inter-rater reliability.

### 2.4 Statistical analysis

All data were analysed with SPSS Statistics version 26 (IBM Corporation, Armonk, New York, USA). P-values < 0.05 were considered to represent a statistically significant result. Univariate linear regression analysis was performed for low and high-risk groups to evaluate the relationship between the regional T1 and T2 relaxation times and PMA. Mann-Whitney U tests for continuous and chi-square tests for categorical variables were used to identify differences in clinical characteristics between both groups. Analysis of covariance (ANCOVA) tests were used to determine the effect of high- versus low-risk on relaxation parameters, covarying for PMA. When significant differences were found between both risk groups, ANCOVA tests, covarying for PMA, were used to explore differences in relaxation times in subgroups, including extreme preterm birth (<28 weeks), bronchopulmonary dysplasia, severe brain injury, postnatal steroids, and prolonged ventilation (>8 days). Furthermore, the discriminative ability to classify high-risk patients based on relaxation times was assessed using receiver operating characteristic (ROC) curves and evaluated with the area under the ROC curves (AUC). AUC between 0.7 and 0.8 was considered acceptable, 0.8 to 0.9 was considered excellent (Mandrekar, 2010).

The interaction effect of PMA and risk were analysed using regression analysis to determine whether the slope of age-related decrease of relaxation times around TEA was different for high and low-risk groups.

Intraclass correlation coefficients (ICC) were used to assess the inter- and intra-rater variability of manually drawn regions of interest. ICC were calculated in SPSS using a two-way mixed model with absolute agreement measures. Correlation coefficients were interpreted as poor (<0.40), fair (0.40–0.59), good (0.60-0.74) or excellent (0.75–1.0) agreement (Cicchetti, 1994). Graphs were prepared with GraphPad Prism version 8 (GraphPad Software, San Diego, California USA).

## 3 Results

### 3.1 Study participants

Of the 91 patients, 21 patients were excluded from the analyses due to excessive movement in the synthetic dataset (n = 7), failed watershed skull-stripping or segmentation errors by MANTiS (n = 12), or corrupted synthetic DICOM dataset (n = 1). One patient was excluded due to poor coregistration of the segmentation by MANTiS with the synthetic dataset. The resulting study cohort consisted of 70 neonates, of which 39 were male (56%). Mean gestational age at birth was 29.1 weeks ± 2.4 standard deviations (SD). Mean gestational age at birth was higher in the low-risk group (30.0 weeks ± 1.5 SD) compared to the high-risk group (27.0 weeks ± 2.6 SD, p < .001). Birth weight (+303 g) and head circumference at birth (+2.5 cm) were also greater in the low-risk group (p < 0.001). There was no significant difference in sex (p = .89), birth weight Z-score (p = .22), and PMA at scan (p = .29) between high- and low-risk groups (Table 1). The majority of the high-risk infants (19/22; 86%) were born extremely preterm (≤28 weeks gestation).

**Table 1:** Demographic characteristics.

	Total (n = 70)	High-risk (n = 22)	Low-risk (n = 48)	Difference (p-value)
*Continuous parameters*				
Gestational age (wk) *	29.1 (2.4)	27.0 (2.6)	30.0 (1.5)	<0.001
PMA (wk) *	39.9 (0.8)	39.8 (0.7)	40.0 (0.8)	0.29
Birth weight (g) *	1262.5 (375.9)	959.8 (378.9)	1401.3 (284.1)	<0.001
Birth weight Z-score *	0.04 (0.68)	0.21 (0.67)	0.03 (0.67)	0.22
Head circumference at birth (cm) °	27.0 (25–29)	24.5 (22–26)	27.6 (26–29)	<0.001
*Categorical parameters*				
Sex (male)	39/70 (56%)	12/22 (55%)	27/48 (56%)	0.89
Extremely preterm (<28 weeks)	24/70 (34%)	19/22 (86%)	3/22 (14%)	<0.001
Intra-uterine growth restriction	6/70 (9%)	2/22 (9%)	4/48 (8%)	0.92
*Germinal matrix hemorrhage*				
Grade I-II	12/70 (17%)	6/22 (27%)	6/48 (13%)	0.13
Grade III	1/70 (1%)	1/22 (4%)	*–*	
Grade IV	1/70 (1%)	1/22 (4%)	*–*	*–*
Postnatal steroids	7/70 (10%)	7/22 (32%)	*–*	*–*
Surgery	6/70 (9%)	6/22 (27%)	*–*	*–*
Significant brain injury	3/70 (4%)	3/22 (14%)	*–*	*–*
Bronchopulmonary dysplasia				*–*
Mild	4/70 (6%)	4/22 (18%)	*–*	*–*
Moderate	8/70 (11.4%)	8/22 (32%)	*–*	*–*
Severe	7/70 (10%)	7/22 (32%)	*–*	*–*

* Data are mean (±standard deviation).

° Data are median (interquartile range).

PMA: postmenstrual age.

### 3.2 Inter- and intra-rater reliability

The results of reproducibility for the manually drawn regions of interests are presented in inline SupplementaryTable 2. Intraclass coefficients (ICC) for intra-rater reliability were good or excellent for all relaxation time measurements (ICC average range 0.67–0.88). Inter-rater reliability was good or excellent as-well for all measurements (ICC range 0.62–0.85), except for the frontal white matter, where it was fair (ICC 0.46).

### 3.3 Relationship between regional relaxation time and PMA

**Fig. 3:**
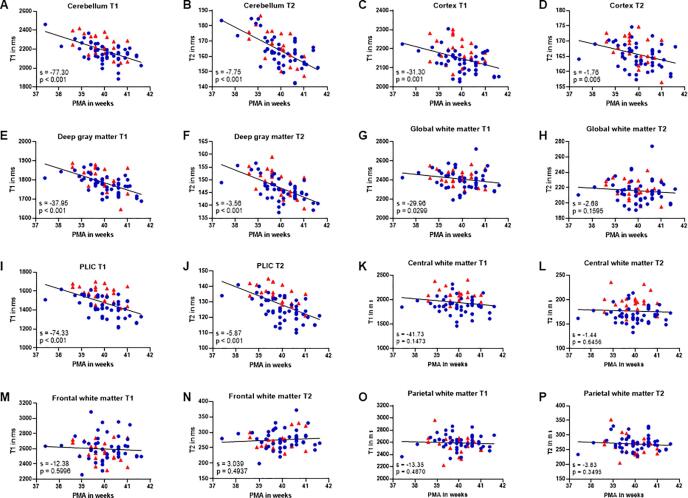
Relationship between synthetic MRI relaxation times and PMA around term equivalent age (range 37–42 weeks). Regression lines are shown for all subjects. The low-risk group is depicted as blue circles and the high-risk group as red triangles. s: slope in ms/week; PLIC: posterior limb of internal capsule; PMA: postmenstrual age at scan. (For interpretation of the references to colour in this figure legend, the reader is referred to the web version of this article.)

Regression analysis was performed on data of all subjects to determine the relationship between synthetic MRI relaxation times and PMA around TEA (range 37–42 weeks). A linear age-related decrease of T1 and T2 relaxation times was found in all brain tissue segments. However, this decrease was only statistically significant (p < .005) in the deep gray matter, the cerebellum, the cortex, and the PLIC (Fig. 3). The age-related decrease of relaxation times was most evident in the cerebellum (slope = −77.3 and −7.75 ms/week for T1 and T2 respectively, p < .001) and the PLIC (slope = −74.33 and −5.87 ms/week, p < .001). The relaxation times in the mainly unmyelinated global and regional white matter did, on the other hand, not display a significant age-dependent correlation with PMA between 37- and 42-weeks PMA. The results of the regional linear regressions for the low- and high-risk groups are shown in detail in Table 2. The regional correlations in the high-risk group were generally weaker compared to the low-risk group. However, the interaction terms between PMA and risk revealed no significant difference in slope between the high and low-risk groups (Table 3).

**Table 2:** Linear regression of T1 and T2 relaxation times in function of postmenstrual age (PMA) for low- and high-risk groups.

	B†		β		R^2^adj		*P*-value	
	LR	HR	LR	HR	LR	HR	LR	HR
*T1 relaxation time*								
Cortex	−29.6 (9.1)	−23.3 (20.8)	−0.43	−0.24	0.17	0.01	0.002*	0.28
Deep gray matter	−33.0 (6.7)	−47.8 (16.2)	−0.58	−0.55	0.32	0.27	< 0.001*	0.008^(*)^
Cerebellum	−69.2 (14.9)	−86.2 (30.6)	−0.57	−0.53	0.30	0.25	< 0.001*	0.01^(*)^
Global WM	−21.9 (18.2)	−28.3 (24.8)	−0.18	−0.25	0.01	0.01	0.24	0.27
PLIC	−68.8 (15.1)	−56.4 (24.0)	−0.56	−0.47	0.30	0.18	< 0.001*	0.03^(*)^
Central WM	−31.3 (28.8)	−8.0 (45.4)	−0.16	−0.04	0.00	−0.05	0.28	0.86
Frontal WM	−3.9 (28.5)	−55.3 (41.1)	−0.02	−0.29	−0.02	0.04	0.89	0.19
Parietal WM	3.3 (20.4)	−64.4 (47.3)	0.02	−0.29	−0.02	0.04	0.87	0.19
*T2 relaxation time*								
Cortex	−1.3 (0.7)	−2.9 (1.3)	−0.27	−0.44	0.05	0.15	0.06	0.04^(*)^
Deep gray matter	−3.1 (0.6)	−4.4 (1.3)	−0.60	−0.60	0.35	0.33	< 0.001*	0.003^(*)^
Cerebellum	−6.9 (1.4)	−10.1 (2.6)	−0.59	−0.66	0.33	0.41	< 0.001*	0.001^(*)^
Global WM	−1.1 (2.7)	−3.4 (3.9)	−0.06	−0.20	−0.02	−0.01	0.70	0.38
PLIC	−5.3 (1.1)	−5.4 (1.6)	−0.59	−0.61	0.33	0.34	< 0.001*	0.003^(*)^
Central WM	−0.3 (2.9)	1.7 (6.4)	−0.01	0.06	−0.02	−0.05	0.93	0.80
Frontal WM	1.9 (5.2)	6.6 (9.0)	0.05	0.16	−0.02	−0.02	0.72	0.47
Parietal WM	−0.01 (4.2)	−15.9 (10.2)	0.00	−0.33	−0.02	0.06	> 0.99	0.13

LR: low-risk group, HR: high-risk group, WM: white matter.

†Data are means ± standard deviations.

^(*)^p < .05 is defined as the significance level.

**Table 3:** Analysis of covariance (ANCOVA) of relaxation times between low- and high-risk groups, covarying for postmenstrual age (PMA) at scan.

	Low-risk group RT in ms†	High-risk groupRT in ms†	Interaction p-value††	Difference (Δ%)	*P*-value for difference
*T1 relaxation*					
Cortex	2135 ± 8 (2133)	2185 ± 12 (2188)	0.76	2.30%	0.001(*)
Cerebellum	2168 ± 13 (2164)	2236 ± 19 (2245)	0.6	3.10%	0.005(*)
Deep gray matter	1779 ± 6 (1777)	1805 ± 9 (1810)	0.03	1.4%	0.03(*)
Global WM	2402 ± 14 (2400)	2427 ± 21 (2429)	0.86	1.00%	0.33
PLIC	1439 ± 12 (1435)	1571 ± 18 (1578)	0.69	9.20%	< 0.001(*)
Central WM	1876 ± 23 (1874)	2091 ± 34 (2095)	0.69	11.50%	< 0.001(*)
Frontal WM	2617 ± 22 (2613)	2564 ± 33 (2566)	0.36	−1.90%	0.21
Parietal WM	2604 ± 19 (2603)	2560 ± 28 (2561)	0.15	−1.70%	0.2
*T2 relaxation*					
Cortex	165 ± 1 (1 6 5)	167 ± 1 (1 6 7)	0.25	1.10%	0.07
Cerebellum	163 ± 1 (1 6 3)	165 ± 2 (1 6 6)	0.29	1.20%	0.35
Deep gray matter	146 ± 1 (1 4 6)	149 ± (1 4 9)	0.34	1.50%	0.02(*)
Global WM	215 ± 2 (2 1 5)	218 ± 3 (2 1 8)	0.65	1.40%	0.42
PLIC	126 ± 1 (1 2 5)	134 ± 1 (1 3 5)	0.97	6.90%	< 0.001(*)
Central WM	170 ± 3 (1 7 0)	193 ± 4 (1 9 2)	0.77	13.40%	< 0.001(*)
Frontal WM	276 ± 4 (2 7 6)	275 ± 6 (2 7 5)	0.66	−0.40%	0.89
Parietal WM	273 ± 4 (2 7 3)	263 ± 6 (2 6 4)	0.11	−3.70%	0.16

PLIC: posterior limb of the internal capsule, WM: white matter, RT: relaxation time.

† Data are means ± standard deviations; unadjusted means are given in brackets.

†† interaction effect between risk group and postmenstrual age to assess the homogeneity of regression slopes for low- and high-risk groups.

(*) p < .05 is defined as the significance level

### 3.4 Regional relaxation times in low- and high-risk groups

**Fig. 4:**
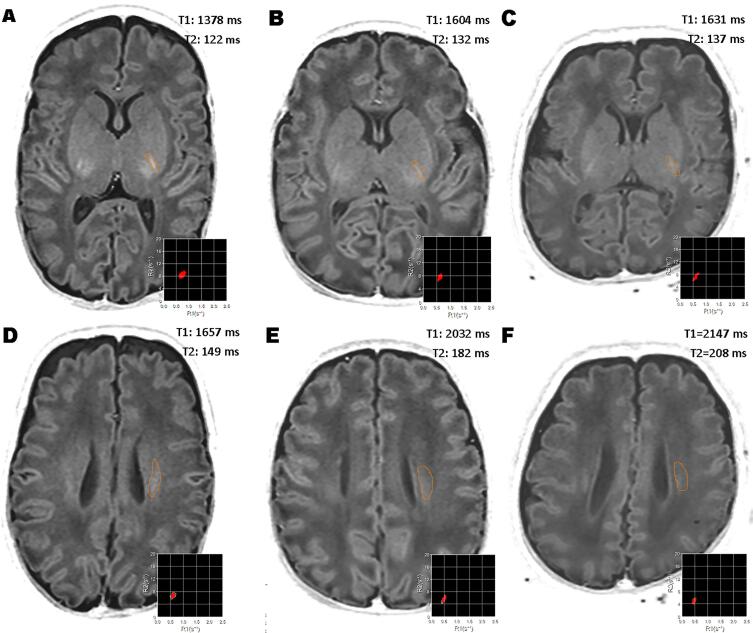
A representative sample of synthetic T1 PSIR images of a low-risk patient (A,D), and two high-risk patients (B,E and C,F). All MR studies were previously reported normal. Although all the images have a similar appearance, the T1 and T2 relaxation times in the posterior limb of the internal capsule (PLIC) and central white matter are much longer in both high-risk patients (B,E and C,F). Upper row: region of interest in the PLIC. Bottom row: region of interest in the central white matter of the centrum semiovale.

An analysis of covariance (ANCOVA) for both risk groups, adjusted for PMA, was used to evaluate the impact of severe postnatal morbidity in the high-risk group on the T1 and T2 relaxation times (Table 3). Delayed relaxation in the high-risk group was clearly illustrated in the central white matter of the centrum semiovale (T1 Δ = 11.5%, T2 Δ = 13.4%, p < .001) and in the PLIC (T1 Δ = 9.2%, T2 Δ = 6.9%, p < .001) as shown in Fig. 4. ANCOVA also revealed delayed T1 relaxation in the high-risk group in the cerebellum (Δ = 3.1%, p = .005), the cortex (Δ = 2.3%, p = .001), and the deep gray matter (Δ = 1.4%, p = .03). A similar delay of T2 relaxation for the high-risk group compared to the low-risk group without severe morbidity was observed in the deep gray matter (Δ = 1.5%, p = .02), but not in the cerebellum (p = .35) or the cortex (p = .07).

The relaxation times in frontal (T1Δ = −1.9%, p = .21, T2Δ = −0.4%, p = .89) and parietal (T1Δ = −1.7%, p = .20, T2Δ = −3.7%, p = .16) white matter were shorter for the high-risk group, but these differences were not significant.

**Fig. 5:**
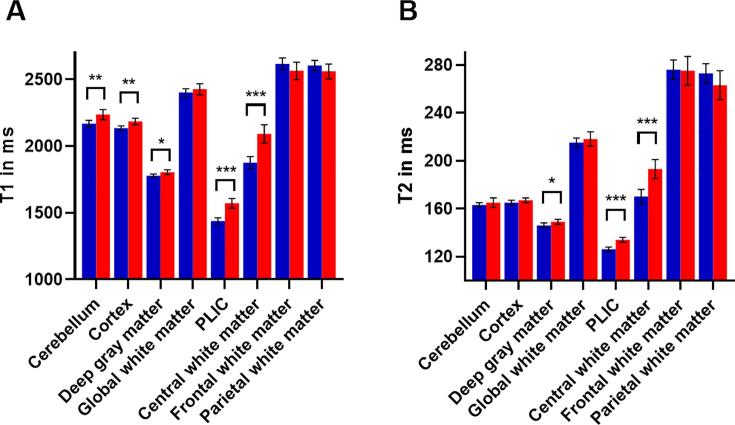
Differences in corrected means between low- and high-risk groups, adjusted for postmenstrual age. PLIC: posterior limb of the internal capsule. Error bars represent ± two standard deviations. The low-risk group is displayed in blue and the high-risk group in red. *p < 0.05, **p < 0.01, ***p < 0.001. (For interpretation of the references to colour in this figure legend, the reader is referred to the web version of this article.)

The relative difference between the regional relaxation times in the low- and high-risk groups are shown in Fig. 5. The relaxation times were shortest in the PLIC for both groups (T1 = 1439 ± 12 ms, T2 = 126 ± 1 ms, mean ± standard deviation), followed by the deep gray matter (T1 = 1779 ± 6 ms, T2 = 146 ± 1 ms). The largely unmyelinated frontal (T1 = 2617 ± 22 ms, T2 = 276 ± 4 ms) and parietal (T1 = 2604 ± 19 ms, T2 = 273 ± 4 ms) white matter demonstrated the most extended relaxation times.

### 3.5 Regional relaxation times in clinical subgroups

ANCOVA tests, varying for PMA, were used to further explore the variations in relaxation times for different clinical subgroups, including neonates with a history of extremely preterm birth (<28w), bronchopulmonary dysplasia, severe brain damage, postnatal steroids, or prolonged mechanical ventilation (>8d). The difference between the presence or absence of each clinical risk factor is shown in Table 4.

**Table 4:** Analysis of covariance (ANCOVA) of relaxation times between clinical subgroups.

	Cortex	*p*	Deep gray matter	*p*	Cerebellum	*p*	PLIC	*p*	Central WM	*P*
*T1 relaxation (ms)*										
Severe brain injury (4/70)	2181 ± 30 *(+1.5%)*	*0.30*	1797 ± 22 *(+0.6%)*	*0.64*	2228 ± 47*(+1.9%)*	*0.41*	1609 ± 50 *(+10%)*	0.009*	2161 ± 90 *(+12.0%)*	0.015*
BPD (21/70)	2191 ± 13 *(+2.7%)*	<0.001*	1808 ± 10 *(+1.7%)*	0.02*	2245 ± 21*(+3.5%)*	0.002*	1565 ± 21 *(+8.1%)*	<0.001*	2084 ± 38 *(+10.3%)*	<0.001*
Postnatal steroids (7/70)	2207 ± 22 *(+2.3%)*	0.008*	1789 ± 17 *(+0.6%)*	0.92	2240 ± 35 *(+2.6%)*	*0.13*	1558 ± 38 *(+5.9%)*	0.04*	2118 ± 68 *(+10.1%)*	0.008*
Prolonged ventilation (11/70)	2192 ± 17 *(+2.3%)*	0.01*	1796 ± 14*(+0.6%)*	*0.47*	2249 ± 27*(+3.3%)*	0.02*	1578 ± 29 *(+7.9%)*	<0.001*	2100 ± 53 *(+9.7%)*	*0.002*
Extremely preterm (24/70)	2178 ± 12 *(+2.0%)*	0.005*	1807 ± 9 *(+1.7%)*	0.01*	2224 ± 19 *(+2.4%)*	0.03*	1553 ± 18 *(+7.7%)*	<0.001*	2054 ± 35 *(+8.4%)*	<0.001*
*T2 relaxation (ms)*										
Severe brain injury (4/70)	167 ± 3 *(+0.6%)*	*0.51*	146 ± 4 *(−)*	*0.60*	164 ± 10 *(+0.6%)*	*0.64*	134 ± 2 *(+3.9%)*	*0.14*	202 ± 9 *(+15.4%)*	0.01*
BPD (21/70)	167 ± 5 *(+1.2%)*	*0.09*	149 ± 5 *(+2.1%)*	0.02*	167 ± 11 *(+%)*	*0.25*	135 ± 7 *(+7.1%)*	<0.001*	192 ± 18 *(+12.3%)*	<0.001*
Postnatal steroids (7/70)	165 ± 2 *(−)*	*0.71*	147 ± 2 *(−)*	*0.58*	162 ± 3 *(−)*	*0.50*	132 ± 3 *(+3.1%)*	*0.12*	203 ± 17 *(+16.7%)*	<0.001*
Prolonged ventilation (11/70)	166 ± 5 *(−)*	*0.52*	147 ± 1 *(−)*	*0.86*	164 ± 2 *(−)*	*0.99*	134 ± 2 *(+5.6%)*	0.006*	198 ± 6 *(+14.5%)*	<0.001*
Extremely preterm (24/70)	167 ± 1 *(+1.2%)*	*0.12*	149 ± 1 *(+2.1%)*	0.02*	164 ± 2 *(−)*	*0.58*	133 ± 1 *(+5.6%)*	<0.001*	189 ± 4 *(+10.5%)*	<0.001*

Analysis of covariance (ANCOVA), covarying for postmenstrual age (PMA) at scan. Data are mean ± standard error. Only the relaxation times of the positive groups are shown. Differences between positive and negative groups are given in brackets. BPD: bronchopulmonary dysplasia, PLIC: posterior limb of the internal capsule, WM: white matter *p* < .05 is defined as the significance level

Analogous to the previous findings in the high and low-risk groups, the clinical subgroups' most considerable differences were found in the PLIC and the central white matter. In the central white matter, the relaxation time prolongation was significant for all clinical risk factors. The differences were slightly more pronounced in the T2 measurements (average + 13.9%; range 10.5%-16.7%) than in the T1 measurements (average + 10.1%; range 8.4%–12%). The differences in relaxation times in the PLIC were also substantial but were more pronounced in the T1 measurements (average + 7.9%) than in the T2 measurements (average + 5.1%).

The differences were found to be less pronounced in the relaxation time measurements of the cortex (≤2.7%), the deep gray matter (≤1.6%), and the cerebellum (≤3.6%).

### 3.6 Risk group classification based on tissue relaxation times

**Fig. 6:**
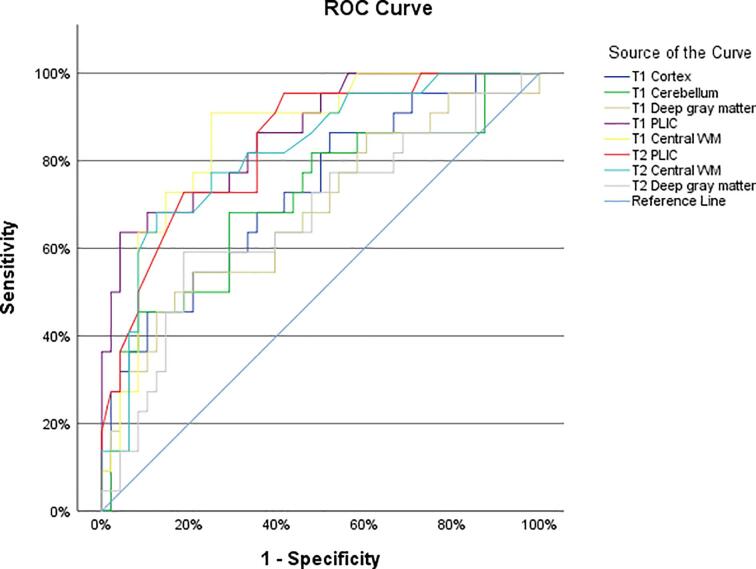
Receiver operator curves demonstrate the diagnostic ability to classify high-risk patients based on regional T1 and T2 relaxation time measurements. PLIC: posterior limb of the internal capsule, WM: white matter.

Whether high-risk patients could be distinguished from low-risk patients based solely on their regional tissue relaxation times was assessed by receiver operating characteristic (ROC) curves (Fig. 6) and evaluated by the area under the curve (AUC) of ROC curves (Table 5). The discriminating diagnostic abilities of both T1 and T2 relaxation measurements were found to be excellent in the PLIC and the central white matter (AUC range 0.82–0.86), acceptable for the T1 measurements in the cortex and the cerebellum (AUC 0.73 and 0.71), and insufficient for T1 and T2 relaxation time measurements in the deep gray matter (AUC 0.68 and 0,67 respectively) (Mandrekar, 2010).

**Table 5:** Area under the receiver operating characteristics (ROC) curve.

	AUC	95% CI	*p*-value
T1 Cortex	0.727	0.600–0.855	0.002
T1 Cerebellum	0.710	0.573–0.848	<0.005
T1 Deep gray matter	0.684	0.543–0.824	0.014
T1 PLIC	0.864	0.774–0.954	<0.001
T1 Central WM	0.856	0.765–0.946	<0.001
T2 PLIC	0.839	0.741–0.936	<0.001
T2 Central WM	0.856	0.713–0.936	<0.001
T2 Deep gray matter	0.665	0.521–0.808	0.028

Area under the ROC curve (AUC) analysis to distinguish high-risk patients from low-risk patients, based on T1 and T2 relaxation time measurements. CI: Confidence interval. Only the regions with significant differences between high- and low-risk groups were included. WM: white matter, PLIC: posterior limb of the internal capsule.

## 4 Discussion

Our results demonstrate that synthetic MRI-based relaxometry can detect age-dependent variability in very preterm born infants, even when the infants are scanned at close to TEA (range 37–42 weeks gestational age). In the developing brain, T1 and T2 relaxation are strongly influenced by microstructural changes in bulk water content and compartmentalization, associated with brain maturation and myelination. According to the limited number of studies on relaxation times in very preterm born infants (Counsell et al., 2003, Knight et al., 2018, Maitre et al., 2014, Schneider et al., 2016, Williams et al., 2005), brain maturation was associated with a linear decline of T1 and T2 relaxation times, as was clearly illustrated in the cortex, the deep gray matter, and in the PLIC. Our study adds the fact that there is a linear age-dependent relationship in the cerebellum. The age-dependent relaxation time decline was most pronounced in the PLIC and the cerebellum. Both structures undergo rapid growth in the last weeks of the third trimester (Dubois et al., 2014) and are strongly correlated with neurodevelopmental disabilities when their development is disrupted (Ment et al., 2009). The dependence of T1 and T2 relaxation times on PMA implies that PMA should be taken into account before any interpretation of the relaxometry data of neonatal brains, even when standardized around TEA.

In contrast to earlier reports (Counsell et al., 2003, Leppert et al., 2009, Nossin-Manor et al., 2013, Schneider et al., 2016, Thornton et al., 1999), except for the already myelinated PLIC, the decrease of T1 and T2 relaxation times was not significant in the global or regional white matter in our subjects. However, these previous studies evaluated trends over a more extended period, which often included only a few scans near TEA. Since the progression of myelination is a tightly regulated temporal and regional process, these long-term trends may differ from those in a more restricted timeframe, as was the case in our study (Yakovlev and Lecours, 1967).

We investigated for the first time the impact of neonatal morbidity on relaxation values at TEA. The rate of age-related decrease in relaxation times around TEA was not significantly affected by severe postnatal morbidity. However, compared to low-risk infants, patients with severe postnatal morbidity and thus at high-risk of neurodevelopmental impairment demonstrated prolonged T1 and T2 relaxation in central white matter, PLIC, cerebellum, cortex, and deep gray matter. This difference was most pronounced in the central white matter of the centrum semiovale and the PLIC. These are both white matter structures that are already partially myelinated at TEA, and several studies have shown that diffusion tensor imaging parameters of centrum semiovale and PLIC at term equivalent MRI scans are correlated with long term neurodevelopmental outcome (Ment et al., 2009). The frontal and parietal white matter, on the other hand, are only sparsely myelinated at term, and observed relaxation times were not significantly different in the high-risk group compared to low-risk infants without complicated postnatal clinical course. Therefore, this finding supports the common concept that early and rapidly myelinating white matter structures may be more susceptible to brain injury in early postnatal life. Alternatively, this could indicate that injury to oligodendrocyte progenitors and disrupted maturation of myelin-forming oligodendrocytes may only become apparent at the time of true myelination (Back et al., 2007). Although the cerebellar myelination precedes the myelination of the supratentorial structures such as the PLIC, the effect of neonatal morbidity was less pronounced compared to the PLIC and the centrum semiovale. However, supratentorial white matter is particularly susceptible to axonal injury, while cerebellar injury is commonly marked by growth failure (Ment et al., 2009, Volpe, 2009). Furthermore, the automatic segmentation of the cerebellum by MANTiS included both cortical and white matter structures, while the PLIC and the centrum semiovale only represent major white matter fiber bundles.

The application of conventional MRI to predict the outcome of very preterm born infants is limited because it cannot quantify microstructural brain injury (Counsell, 2003). There is a growing need for quantitative biomarkers to identify high-risk patients for early neurorehabilitation (Parikh, 2016). Synthetic MRI has the unique property of combining excellent diagnostic neonatal MRI imaging (Schmidbauer et al., 2020, Vanderhasselt et al., 2020) with potent biomarkers such as relaxation times and robust volumetrics (Vanderhasselt et al., 2020), which are usually only available in research centers and require considerable postprocessing analyses. These quantitative data might also support machine learning methods, as was previously demonstrated in hereditary diffuse leukoencephalopathy with spheroids and multiple sclerosis (Mangeat et al., 2020). Because conventional image reconstruction, volumetrics, and relaxometry are all based on a single 6-minute scan sequence, synthetic MRI can be easily integrated into routine neonatal scanning protocols.

The following limitations merit consideration. First, since this was a single-center retrospective study performed on a single MRI system, it was not possible to verify the repeatability of synthetic relaxometry acquisition or the generalization of the results to other systems. A previous study, however, demonstrated no significant differences in T1 and T2 relaxation times in subsequent acquisitions using different head coils (Krauss et al., 2015). Second, quantitative T1 and T2 values may vary according to the technique used and are therefore not directly comparable in absolute value between different studies (Lee et al., 2018). However, this bias should be systematic between different methods, and the results should be reproducible on other scanners. Third, although it was feasible to identify high-risk patients with excellent accuracy based on relaxation times in the PLIC and the central white matter, not all high-risk infants will later present with neurodevelopmental disabilities. Finally, comparison with healthy term-born children or serial imaging was not performed in this study.

Brain maturation values in healthy children were recently reported using synthetic MRI relaxometry from childhood to adolescence (Lee et al., 2018). Future studies should aim to build a database that allows the analysis of the correlation of relaxometry data of preterm infants with neurodevelopmental outcome. The increased use of quantitative synthetic MRI in clinical practice can facilitate and promote the use and understanding of the value of these potential biomarkers.

## 5 Conclusion

Synthetic MRI-based relaxometry measurements in the preterm brain were sensitive to age-related maturational changes close to TEA. We observed significantly prolonged regional T1 and T2 relaxation times in neonates with severe postnatal morbidity. Future studies are needed to connect these findings with long-term outcomes and to determine whether these synthetic quantitative tissue maps can serve as quantitative biomarkers. Synthetic MRI is easily integrated into clinical neonatal scan protocols and offers an excellent opportunity to combine qualitative diagnostic imaging with volumetric and quantitative relaxometry, ideally suited for further research and the new era of artificial intelligence.

## CRediT authorship contribution statement

**Tim Vanderhasselt:** Conceptualization, Methodology, Formal analysis, Writing - original draft. **Roya Zolfaghari:** Formal analysis, Writing - original draft, Software. **Maarten Naeyaert:** Conceptualization, Software. **Jeroen Dudink:** Conceptualization. **Nico Buls:** Methodology, Formal analysis. **Gert-Jan Allemeersch:** Conceptualization, Formal analysis. **Hubert Raeymakers:** Conceptualization, Methodology. **Filip Cools:** Conceptualization. **Johan Mey:** Supervision, Conceptualization.
