# Supplementary material for: Synthetic MRI demonstrates prolonged regional relaxation times in the brain of preterm born neonates with severe postnatal morbidity
Source: Neuroimage Clin. 2020 Dec 24;29:102544. doi: 10.1016/j.nicl.2020.102544 (PMC7786121; doi:10.1016/j.nicl.2020.102544)
Supplement: Supplementary data 1 [file mmc1.docx]

**Supplemental material**

**Inline Supplementary Table 1** - MRI Acquisition parameters (3T Philips scanner)

|  | Synthetic MRI | Sag 3D T2WI | Sag 3D T1WI | SWI | DWI | Multishell DTI |
| --- | --- | --- | --- | --- | --- | --- |
| FoV (mm) | 181 x 150 x 105 | 160 x 160 x 180 | 175 x 175 x 110 | 150 x 124 x 100 | 180 x 180 x 139 | 200 x 200 x 100 |
| Voxel size (mm) | 0.7 x 0.9 x 3.0 | 0.9 x 0.9 x 0.9 | 1.0 x 1.0 x 1.0 | 0.6 x 0.6 x 2.0 | 1.5 x 1.9 x 4 | 2.0 x 2.0 x 2.0 |
| Slice gap (mm) | 0.3 | - | - | - | 1 | 0 |
| TR (ms) | 5000 | 2500 | 6.076 | 31 | 3870 | 6766 |
| TE (ms) | 13 / 100 | 331 | 2.729 | 7.2 | 103 | 106 |
| TI (ms) | 4 TI's (default) | - | 800 | - | - | 220 |
| ETL (ms) | 10 | 117 | 141 | - | - | - |
| SENSE factor | 1.7 | 2 / 2.5 | 1 | 2 / 1 | 2 | 2.2 |
| Bandwitdh (kHz) | 220.5 | 457 | 298 | 254 | 1436 | 1913 |
| Averages | 1 | 1 | 1 | 1 | 1 | 1 |
| Flip angle (degrees) | 90 | 90 | 8 | 17 | 90 | 90 |
| Scan time (min:sec) | 6:10 | 2:00 | 3:37 | 2:42 | 0:46 | 16:34 |

**Inline Supplementary Table 2** - Intra-rater and inter-rater reliability.

|  | ICC intra-rater (n=70/70) | ICC inter-rater (n=35/70) |
| --- | --- | --- |
| **T1 Relaxation** |  |  |
| PLIC | .88 (.81 - .93) | .76 (.61 - .86) |
| Frontal WM | .76 (.63 - .84) | .47 (.12 - .72) |
| Parietal WM | .78 (.67 - .86) | .62 (.44 - .77) |
| Central WM | .78 (.66 - .86) | .70 (.51 - .83) |
| **T2 Relaxation** |  |  |
| PLIC | .78 (.66 - .86) | .85 (.76 - .92) |
| Frontal WM | .67 (.51-.78) | .46 (.18 - .68) |
| Parietal WM | .83 (.74 -.89) | .64 (.45 - .78) |
| Central WM | .77 (.65 - .86) | .72 (.60 - .84) |

ICC: intraclass correlation coefficient, using a two-way mixed model with absolute agreement measures. Brackets represent 95% confidence intervals.
